# Diagnostic accuracy of perfusion-weighted phase-resolved functional lung magnetic resonance imaging in patients with chronic pulmonary embolism

**DOI:** 10.3389/fmed.2023.1256925

**Published:** 2023-09-26

**Authors:** Jianghui Duan, Sheng Xie, Hongliang Sun, Jing An, Huan Li, Ling Li, Robert Grimm, Andreas Voskrebenzev, Jens Vogel-Claussen

**Affiliations:** ^1^Department of Radiology, Peking University China-Japan Friendship School of Clinical Medicine, Beijing, China; ^2^Department of Radiology, China-Japan Friendship Hospital, Beijing, China; ^3^DL Department, Siemens Shenzhen Magnetic Resonance Ltd., Shenzhen, China; ^4^Department of Nuclear Medicine, China-Japan Friendship Hospital, Beijing, China; ^5^MR Application Predevelopment, Siemens Healthcare GmbH, Erlangen, Germany; ^6^Diagnostic and Interventional Radiology, Hannover Medical School, Hannover, Germany

**Keywords:** chronic pulmonary embolism, lung, perfusion, phase-resolved functional lung, pulmonary hypertension

## Abstract

**Purpose:**

This study aimed to evaluate the diagnostic performance of perfusion-weighted phase-resolved functional lung (PW-PREFUL) magnetic resonance imaging (MRI) in patients with chronic pulmonary embolism (CPE).

**Materials and methods:**

This study included 86 patients with suspected chronic thromboembolic pulmonary hypertension (CTEPH), who underwent PREFUL MRI and ventilation/perfusion (V/Q) single-photon emission computed tomography/computed tomography (SPECT/CT). PREFUL MRI was performed at 1.5 T using a balanced steady-state free precession sequence during free breathing. Color-coded PW images and quantitative parameters were obtained by postprocessing. Meanwhile, V/Q SPECT/CT imaging was performed as a reference standard. Hypoperfused areas in the lungs were scored for each lobe and segment using V/Q SPECT/CT images and PW-PREFUL MR images, respectively. Normalized perfusion (Q_N_) and perfusion defect percentage (QDP) were calculated for all slices. For intra- and interobserver variability, the MRI images were analyzed 2 months after the first analysis by the same radiologist and another radiologist (11 years of lung MRI experience) blinded to the results of the first reader.

**Results:**

Of the 86 enrolled patients, 77 met the inclusion criteria (36 diagnosed with CPE using V/Q SPECT/CT and 41 diagnosed with non-CPE etiology). For the PW-PREFUL MRI, the sensitivity, specificity, accuracy, and positive and negative predictive values for the diagnosis of CPE were 97, 95, 96, 95, and 98% at the patient level; 91, 94, 93, 91, and 94% at the lobe level, and 85, 94, 92, 88, and 94% at the segment level, respectively. The detection of segmental and subsegmental hypoperfusion using PW-PREFUL MRI revealed a moderate agreement with V/Q SPECT/CT (*κ* = 0.65; 95% confidence interval: 0.61–0.68). The quantitative results indicated that the Q_N_ was lower in the CPE group than in the non-CPE group [median score (interquartile range, IQR) 6.3 (2.8–9.2) vs. 13.0 (8.8–16.7), *p* < 0.001], and the QDP was higher [median score (IQR) 33.8 (15.7–51.7) vs. 2.2 (1.4–2.9), *p* < 0.001].

**Conclusion:**

PREFUL MRI could be an alternative test to detect CPE without requiring breath-hold, contrast agents, or ionizing radiation.

## Introduction

Chronic thromboembolic pulmonary hypertension (CTEPH) is a complication of pulmonary embolism (PE) and a major cause of pulmonary hypertension (PH). It is often fatal, especially if left untreated (1–3). The diagnosis of CTEPH is based on 2 components: the presence of chronic pulmonary embolism (CPE) and elevated pulmonary arterial pressure. The current guidelines recommend ventilation/perfusion single-photon emission computed tomography (V/Q SPECT) as the first step in diagnosing CPE (1, 2). V/Q SPECT, when combined with a CT scan offers precise morphological information owing to its higher spatial resolution. Computed tomography pulmonary angiography (CTPA) usually reveals the direct signs of CPE at the lobe and segment levels (4–6). However, CTPA has lower sensitivity than V/Q scanning (7), which may be missed by radiologists with no experience. Besides, both V/Q scanning and CTPA result in exposure to ionizing radiation (8). A few studies reported that dynamic contrast-enhanced (DCE) lung perfusion magnetic resonance imaging (MRI) is a suitable method for detecting parenchymal hypoperfusion caused by CTEPH at the individual level (9, 10). Although contrast-enhanced (CE) magnetic resonance angiography (MRA) can also directly reveal a thromboembolic material, its sensitivity and specificity are lower than those of CTPA (5). Moreover, CE-MRA and DCE-MRI pose risks such as allergic reactions, nephrogenic systemic fibrosis, and gadolinium deposition in the brain and other parts of the body (11–13). Furthermore, a longer breath-hold is required for CE MRI data acquisition.

Bauman et al. introduced a contrast-free, proton-based Fourier decomposition (FD) MRI technique (14) that can assess regional lung perfusion and ventilation during free breathing. FD MRI applies spectral analysis to generate perfusion- and ventilation-weighted images by analyzing periodic signal variations in the lung parenchyma. This approach allows for the simultaneous assessment of regional ventilation and perfusion (14, 15). Recently, a new postprocessing technique known as phase-resolved functional lung (PREFUL) MRI has been developed based on FD, which improves the effective temporal resolution through the image-sorting algorithm (16). Previous studies have demonstrated the feasibility and repeatability of PREFUL MRI in healthy individuals and patients with lung diseases (16–18). Further research has also confirmed the potential of PREFUL MRI to predict clinical outcomes in CTEPH after pulmonary endarterectomy (19). However, data regarding the diagnostic accuracy of CPE using PREFUL MRI are still lacking.

Thus, this study aimed to evaluate the diagnostic performance of PREFUL MRI in patients with CPE. Also, the study aimed to investigate the differences in PREFUL MRI-derived parameters between patients in the CPE and non-CPE groups.

## Materials and methods

### Study participants

This study received approval from the China-Japan Friendship Hospital Ethical Review Board (Medical Ethics No. 2017-24). Written informed consent was obtained from all participants. From June 2020 to September 2022, 86 patients suspected of having CTEPH, who underwent PREFUL MRI and V/Q SPECT/CT within two weeks, were screened and enrolled. The inclusion criteria were as follows: (1) patients presenting with persistent shortness of breath or exercise limitation, who were followed up after standard anticoagulation therapy for at least 3 months for acute PE; and (2) patients having elevated systolic pulmonary arterial pressure (> 60 mm Hg) on transthoracic echocardiogram. The exclusion criteria were as follows: (1) patients with contraindications to MRI, such as an implanted cardiac pacemaker, claustrophobia, and metallic foreign matters, in the chest; (2) interval between PREFUL MRI and V/Q SPECT/CT of more than 2 weeks; and (3) severe image artifacts in PREFUL MRI. Severe image artifacts refer to band artifacts at the edge of both lungs, which lead to decreased image quality in postprocessing maps, as previously described (20). Nine patients were excluded based on these criteria, and finally, 77 patients were included in the analysis. CTEPH was diagnosed according to China’s Guidelines for the Diagnosis and Treatment of Pulmonary Hypertension (2021) (21). Additionally, patients presenting with CPE symptoms but without resting PH were classified as having chronic thromboembolic disease (CTED) (22). Subsequently, the patients included in the study were divided into 2 groups: the CPE group and the non-CPE group.

### SPECT/CT examination

SPECT/CT imaging was performed on a dual-detector SPECT/CT scanner (SymbiaT16, Siemens, Germany) following a 1-day protocol, with ventilation imaging first and then perfusion imaging. ^99m^TcO4^−^ with a high radioactive concentration (>370 MBq/0.1 mL) was injected into a closed device to form Technegas. The patients were instructed to inhale Technegas while in a seated position, and the detector was placed as close to their chests as possible for lung ventilation imaging. In the SPECT perfusion mode, the patients received an intravenous injection of 296 MBq ^99m^Tc-labeled macroaggregated albumin. The acquisition parameters involved the use of 2 detectors, each rotating 180° with respect to the other. A total of 32 projections were collected by each detector, with each projection lasting for 13 s, resulting in 64 projections. The acquisition matrix was 128 × 128 at 1.0 × zoom. The reconstruction was performed using an iterative ordered subset expectation maximization algorithm (4 iterations and 8 subsets) and the coronal, sagittal, horizontal, and three-dimensional (3D) images were obtained. Low-dose CT scanning was performed at the same position after acquiring the tomographic image at the end of the inspiration breath-hold, with the following key parameters: tube voltage = 130 kV; effective tube current–time product = 30 mAs; slice thickness = 2.0 mm; reconstruction increment = 1.0 mm; pitch = 0.8, rotation time = 0.6 s. The average dose–length product for chest LDCT was 107.7 mGy cm, and the average measured effective dose was 1.5 ± 0.2 mSv. The estimated effective radiation dose from V/Q SPECT was 3.0 mSv.

### MRI examination

All participants underwent a lung MRI using a 1.5 T MRI scanner (MAGNETOM Aera, Siemens Healthcare, Erlangen, Germany) equipped with an 18-channel torso-phased array coil and a 24-channel spine coil. The participants were scanned in the supine position with their arms placed next to the body. The PREFUL MRI technique was performed using a 2-dimensional balanced steady-state free precession (bSSFP) sequence during free breathing. The imaging parameters were as follows: Echo time (TE) = 0.4 milliseconds; Repetition time (TR) = 1.1 milliseconds; flip angle = 27.5°; bandwidth = 1,680 Hz/pixel; acquisition matrix = 104 × 128; field of view (FOV) = 500 × 500 mm^2^; slice thickness = 15 mm; and parallel imaging acceleration factor = 2. The PREFUL protocol included 5 coronal slices centered at the level of the tracheal bifurcation, 1 slice after another, to cover the chest volume. Each slice comprised 250 images with a period of 62 s, the total acquisition duration for the PREFUL protocol was approximately 5 min. Additionally, 3D ultrashort echo time (UTE) MRI research sequences were used to acquire anatomical information and assist in locating the perfusion defect area. The 3D UTE sequence with a stack-of-spirals trajectory was implemented at end-expiration during free breathing, with the following key parameters: coronal acquisition plane, TR = 3.85 milliseconds; TE = 0.05 milliseconds, flip angle = 5°; FOV = 480 × 480 mm^2^; slice thickness = 1.5 mm; in-plane resolution = 1.5 × 1.5 mm^2^; and spiral interleaves = 328. The acquisition times varied between 6 min and 7 min according to the respiration pattern of patients.

### PREFUL MRI postprocessing

All bSSFP images were analyzed using stand-alone research software (MRLung 2.2.0; Siemens Healthcare, Erlangen, Germany). The postprocessing for PREFUL MRI was performed as described in a previous study (17), including image registration, segmentation, filtering, and phase sorting. Then, perfusion-weighted (PW) lung maps were extracted for each slice from the full cardiac cycle for further image analysis.

The time-resolved bSSFP images were acquired during free breathing. Hence, all PREFUL datasets were first registered using the freely available Advanced Normalization Tools and a group-oriented registration approach to achieve a fixed respiratory position (23, 24). A semiautomatic segmentation of the lung boundaries was performed using the registered images, with the assistance of a semantic convolutional neural network (25). Manual corrections were applied to include the vast majority of the lung parenchyma and exclude large central lung vessels. Then, images were automatically sorted according to their perfusion phase and interpolated to a full cardiac cycle. An automated phase sorting algorithm searched for the full blood-filled ROI (R_sort_) located inside a central pulmonary artery, the aorta, or inside the heart according to the following steps (17).

1. Both lungs were merged with the mediastinum to form a searching ROI (A_s_).
2. A high-pass filter at 0.75 Hz was applied to all registered images to remove respiration-induced signal variations.
3. A standard deviation map (M_std_) and a temporal maximum intensity projection map (M_MIP_) of all filter images were computed for all voxels within A_s_.
4. All voxels above the 98th percentile of M_std_ and the 98th percentile of M_MIP_ were chosen for R_sort_.
5. As a result, R_sort_ consisted of several voxel clusters. After spatial averaging over the MRI signal of R_sort_, a piecewise sinusoidal fit was applied to the resulting signal time series to obtain the corresponding perfusion phases. To avoid different perfusion phases in the voxel clusters and to improve the sine fit, R_sort_ was iteratively adjusted for optimal phase sorting by comparing the goodness of fit parameter of the sine fit. If the sine fit improves, the cluster will be removed from R_sort_. At least one cluster with the highest fit parameter remained in R_sort_.
6. Finally, images were sorted according to their perfusion phases and interpolated into an equidistant time grid with 15 phases covering one cardiac cycle (15 phases is a good compromise – more phases are difficult to distinguish reliable, and fewer phases may increase the risk of mixing information from different phases. Thus, it is more robust with 15 phases. Also, the nominal temporal resolution of 50 milliseconds, assuming a heart rate of 80 beats per minute).

Further, the PW maps were calculated as follows. For each slice, normalized perfusion (Q_N_) was quantified as a percentage by normalizing the signal of each voxel to the signal of a fully blood-filled voxel obtained from an ROI, as proposed by Kjørstad et al. (26):


$$Q_{N}=\frac{Q}{S_{Blood.}}$$

where Q corresponds to the signal amplitude of the parenchymal signal within the cardiac frequency range, and S_Blood_ is the corresponding signal amplitude of a fully blood-filled voxel. To calculate perfusion defect percentage (QDP) maps, the threshold for healthy Q_N_ values was defined as 2% of the fully blood-filled voxel signal value. Voxels with values below this threshold were identified as perfusion defects. The utilized default value of 2% as a cut-off for the perfusion maps proved reliable in previous publications at 1.5 T (27). While an optimized cut-off value for a specific clinical question or patient cohort may show even better performance for detecting CPE, it would not change the present.

### Qualitative analysis

Two nuclear physicians (L.L. and L.H. each with more than 10 years of experience) who were blinded to the clinical data and other imaging results independently reviewed the V/Q SPECT/CT images. Disagreements were resolved through consensus. One radiologist (J.D.) with 6 years of lung MRI experience, who was blinded to the clinical and V/Q SPECT/CT results, reviewed color-coded PW-PREFUL MRI in conjunction with the UTE images. The V/Q SPECT/CT and PREFUL MRI maps were evaluated using a standard lung segment model, consisting of 18 segments in each participant (10 segments in the right lobe and 8 segments in the left lobe) (28). Image interpretation of two examinations was based on the European Association for Nuclear Medicine (EANM) guidelines (29). In a patient-level analysis, a diagnosis of PE was confirmed by V/Q SPECT/CT if at least one segment or two subsegments of a V/Q mismatch in the hypoperfused region were detected. Similarly, PREFUL MRI was interpreted as positive for PE if a hypoperfused region was detected by PW-PREFUL MRI, or at least one lung segment or two subsegments of a hypoperfused region were found in cases where defects caused by emphysema, pneumonia, and pleural effusion could be excluded using the UTE sequence. In addition, in a lobe- and segment-level analysis, lung perfusion was scored visually for each lobe and each segment using the following semiquantitative classification scoring system: 0 = no hypoperfused area; 1 = subsegmental hypoperfused area; and 2 = segmental hypoperfusion. If the mismatch defect in one lung segment involved more than 75%, it was defined as a segmental hypoperfused area; otherwise, it was defined as a subsegment defect. Perfusion changes due to lung parenchymal pathology (e.g., emphysema or pneumonia) observed in the V/Q SPECT/CT or UTE MRI sequences were not regarded as segmental hypoperfusion due to PE according to the EANM criteria (29). For intra- and interobserver variability assessment of the scores, the data were analyzed 2 months after the first analysis by the same radiologist and an additional radiologist (H.S. with 11 years of MRI experience), who was blinded to the results of the first reader.

### Quantitative analysis

Based on the V/Q SPECT/CT results, the patients were divided into CPE and non-CPE groups. Q_N_ and QDP of all slices were evaluated between the 2 groups.

### Statistical analysis

The continuous data were presented as means ± standard deviations or the median values with 25th and 75th percentiles in parentheses and evaluated using the two-tailed Student *t* test (normally distributed variables) or Mann–Whitney *U* test (nonparametric variables). The categorical data were expressed as frequency (*n*) and percentage (%) and evaluated using the *χ*^2^ or Fisher’s exact test. The diagnostic accuracy was assessed for V/Q SPECT/CT and PW-PREFUL MRI using 2 × 2 contingency tables to calculate sensitivity, specificity, accuracy, and positive and negative predictive values. Cohen kappa (*κ*) coefficients were calculated while evaluating the diagnostic agreement for each lung segment using a classification scoring system. The intra- and interobserver agreement was assessed by calculating the Cohen kappa coefficient. The level of agreement was considered as follows: almost perfect when the *κ* value exceeded 0.8, substantial in the range of 0.60–0.79, moderate in the range of 0.40–0.59, fair in the range of 0.20–0.39, and slight for values below 0.20. The statistical analysis was performed using the software SPSS version 26.0 (IBM Corp., NY, USA) and GraphPad Prism version 9.5.1 (GraphPad Software, CA, USA). A two-sided *p* < 0.05 indicated a statistically significant difference.

## Results

### Participant characteristics

**Figure 1:**
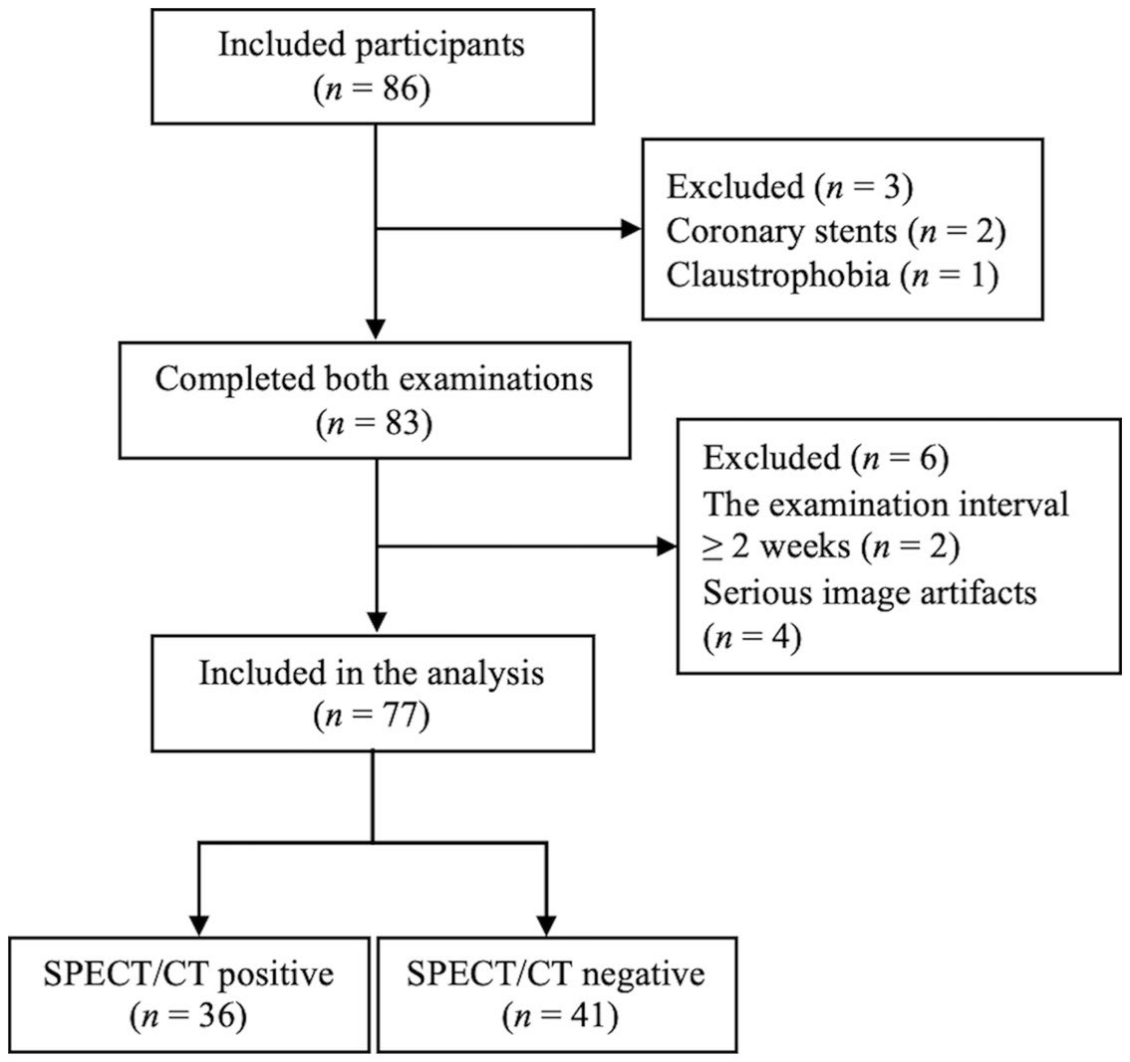
Study flowchart. SPECT/CT, Single-photon emission computed tomography/computed tomography.

Of 77 patients, CPE was diagnosed using V/Q SPECT/CT in 36 patients, including 31 patients with CTEPH and 5 patients with CTED. The remaining 41 patients were diagnosed with non-CPE etiology (Figure 1). The comparison of basic clinical characteristics between the CPE and non-CPE groups is depicted in Table 1. The mean interval between PREFUL MRI and V/Q SPECT/CT was 2.2 ± 2.3 days, with a range of 0–13 days.

**Table 1:** Comparison of basic clinical characteristics between the CPE and non-CPE groups.

Demographic parameter	All patients (*n* = 77)	CPE group (*n* = 36)	Non-CPE group (*n* = 41)	*P*
Age (year)^*^	56 ± 15	53 ± 14	57 ± 17	0.379
Sex (%)				0.001
Female	45 (59)	14 (39)	31 (76)	
Male	32 (41)	22 (61)	10 (24)	
BMI (kg/m^2^) ^*^	24.5 ± 3.5	23.7 ± 2.6	25.2 ± 4.0	0.039
NYHA class				0.006
I	22 (29)	5 (14)	17 (42)	
II	42 (54)	21 (58)	21 (51)	
III	11 (14)	9 (25)	2 (5)	
IV	2 (3)	1 (3)	1 (2)	

Except where indicated, data are numbers of patients, with percentages in parentheses. BMI, Body mass index; CPE, chronic pulmonary embolism; NYHA, New York Heart Association. ^*^Data are means ± standard deviation.

### Diagnostic performance of V/Q SPECT/CT and PW-PREFUL MRI for CPE at the patient, lobe, and segment levels

**Figure 2:**
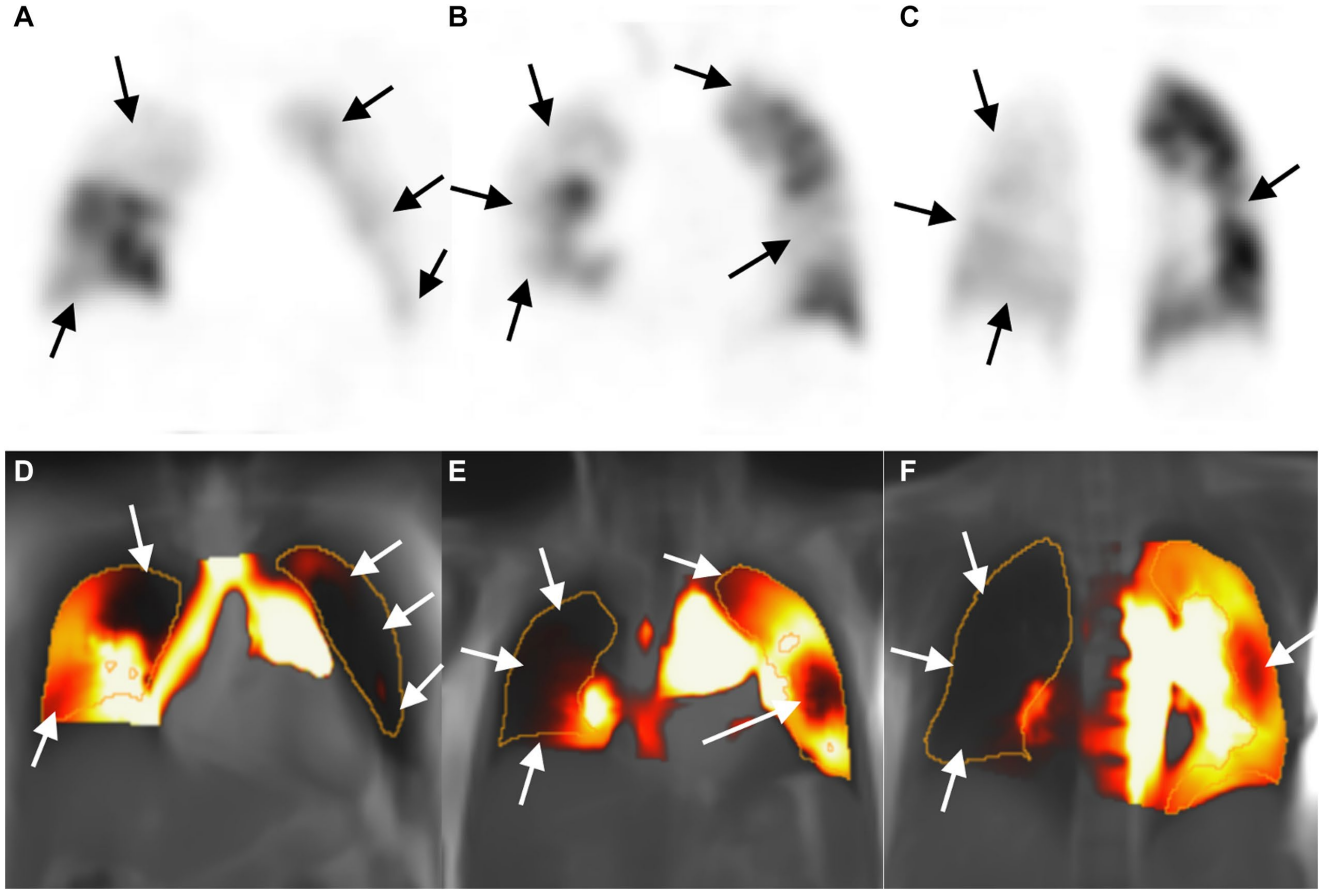
Images of a 53-year-old woman with chronic thromboembolic pulmonary hypertension. Perfusion defect in SPECT perfusion (**A–C**, arrow) and corresponding PW-PREFUL MRI (**D–F**, arrow) located in the whole right lung (except for subsegment IV and segment V) and in the left segments III, IV, and V, and subsegment VI. MRI, Magnetic resonance imaging; PREFUL, phase-resolved functional lung; PW, perfusion-weighted; SPECT, single-photon emission computed tomography.

**Figure 3:**
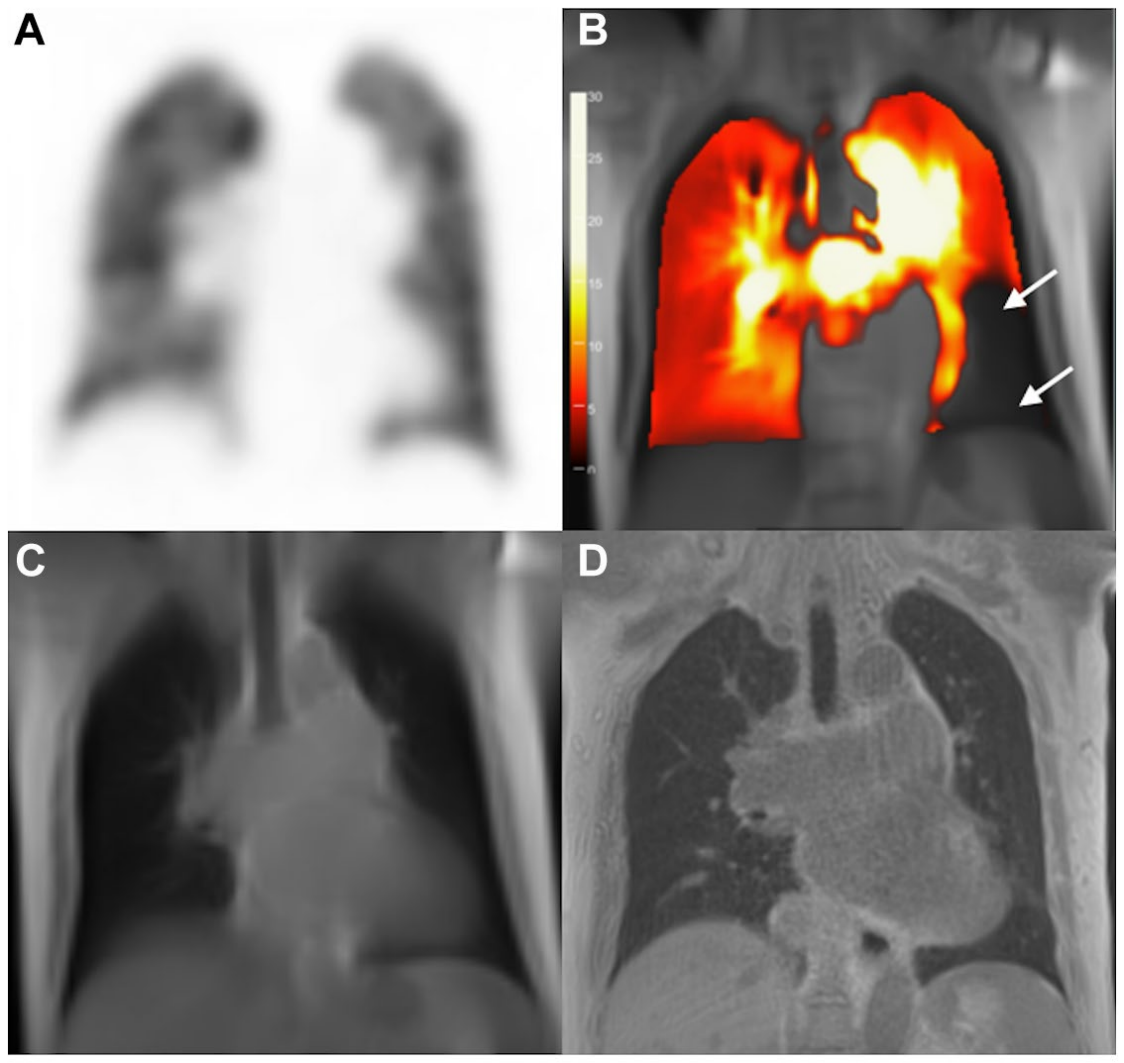
Images of a 75-year-old woman with pulmonary hypertension. SPECT perfusion image **(A)** reveals normal perfusion and corresponding PW-PREFUL MRI **(B)** reveals a false-positive diagnosis for PE. Arrow-marked areas indicate a perfusion defect in the left segment V and segment VII/VIII. **(C)** bSSFP, and **(D)** 3D-UTE; bSSFP, Balanced steady-state free precession; 3D-UTE, 3-dimensional ultrashort echo time; MRI, magnetic resonance imaging; PE, pulmonary embolism; PREFUL, phase-resolved functional lung; PW, perfusion-weighted; SPECT, single-photon emission computed tomography.

With V/Q SPECT/CT results as the reference standard, CPE was diagnosed using PW-PREFUL MRI in 36 patients, as displayed in Figure 2. PREFUL MRI yielded 2 false-positive results (Figure 3) and 1 false-negative result. The sensitivity, specificity, accuracy, and positive and negative predictive values for detecting CPE at the patient, lobe, and segment levels are listed in Table 2.

**Table 2:** Diagnostic performance for detecting or excluding CPE at the patient, lobe, and segment levels.

PREFUL MRI/V/Q SPECT/CT	Detection of hypoperfused areas per patient	Detection of hypoperfused areas per lobe	Detection of hypoperfused areas per segment
Sensitivity	97% (35/36) [85–100]	91% (143/157) [85–95]	85% (364/427) [82–88]
Specificity	95% (39/41) [83–99]	94% (214/228) [90–97]	94% (906/959) [93–96]
PPV	95% (35/37) [82–99]	91% (143/157) [86–94]	88% (364/417) [84–90]
NPV	98% (39/40) [85–100]	94% (214/228) [90–96]	94% (906/969) [92–95]
Accuracy	96% (74/77) [89–99]	93% (357/385) [90–95]	92% (1,270/1386) [90–93]

Data in parentheses are numerators and denominators, with 95% confidence intervals in brackets. MRI, Magnetic resonance imaging; NPV, Negative predictive value; PPV, positive predictive value; PREFUL, phase-resolved functional lung; V/Q SPECT/CT, ventilation/perfusion single-photon emission computed tomography/computed tomography.

### Diagnostic agreement between V/Q SPECT/CT and PW-PREFUL MRI

**Figure 4:**
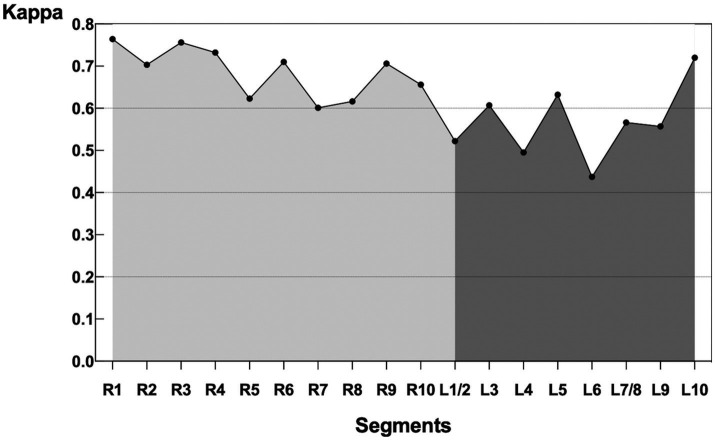
Kappa of each segment comparing PW-PREFUL MRI with V/Q SPECT/CT. For each segment, the level of agreement using the classification scoring system (detecting absent, subsegmental, and segmental hypoperfusion) had a kappa range from 0.44 in the superior segment (L6) of the left lung up to 0.76 in the apical segment (R1) of the right lung. MRI, Magnetic resonance imaging; PREFUL, phase-resolved functional lung; PW, perfusion-weighted; V/Q SPECT/CT, ventilation/perfusion single-photon emission computed tomography/computed tomography.

The kappa value between V/Q SPECT/CT and PW-PREFUL MRI was 0.92 [95% confidence interval (CI): 0.83–1.00], 0.85 (95% CI: 0.80–0.90), and 0.80 (95% CI: 0.77–0.84), respectively, at the patient, lobe, and segment levels. The evaluation of hypoperfused areas for all lung segments using the classification scoring system (detecting absent, subsegmental, and segmental hypoperfusion) revealed a substantial agreement between PW-PREFUL MRI and V/Q SPECT/CT with a kappa of 0.65 (95% CI: 0.61–0.68, Table 3). When considering each segment individually, the level of agreement using the classification scoring system had a kappa range from 0.44 in the superior segment of the left lung up to 0.76 in the apical segment of the right lung, as displayed in Figure 4.

**Table 3:** Consensus diagnosis of PW-PREFUL MRI and V/Q SPECT/CT for all lung segments using the classification scoring system.

PW-PREFUL MRI	V/Q SPECT/CT		
Diagnosis	Absent hypoperfusion	Subsegmental hypoperfusion	Segmental hypoperfusion
Absent hypoperfusion	906	44	9
Subsegmental hypoperfusion	49	89	42
Segmental hypoperfusion	14	73	160

Data are numbers of all lung segments. The Cohen kappa coefficient is 0.65 [95% confidence interval (CI): 0.61–0.68]. MRI, Magnetic resonance imaging; PREFUL, phase-resolved functional lung; PW, perfusion-weighted; V/Q SPECT/CT, ventilation/perfusion single-photon emission computed tomography/computed tomography.

### Intra- and interobserver agreements for PW-PREFUL MRI

The intra- and interobserver agreements for the PW-PREFUL MRI at the patient, lobe, segment, and subsegment levels were almost perfect (kappa 0.80–0.95, Table 4).

**Table 4:** Intra- and interobserver agreements for the PW-PREFUL MRI.

Agreement	Kappa per patient (95% CI)	Kappa per lobe (95% CI)	Kappa per segment (95% CI)	Kappa per segment using the classification scoring system (95% CI)
Intraobserver	0.95 (0.87–1.02)	0.92 (0.87–0.96)	0.90 (0.89–0.93)	0.85 (0.82–0.87)
Interobserver	0.89 (0.79–0.99)	0.86 (0.81–0.92)	0.84 (0.80–0.87)	0.80 (0.77–0.83)

Data in brackets are 95% confidence intervals. CI, Confidence interval; MRI, magnetic resonance imaging; PREFUL, phase-resolved functional lung; PW, perfusion-weighted.

### Quantitative results

Patients in the CPE group had decreased Q_N_ [median (interquartile range, IQR) 6.3 (2.8–9.2)] and increased QDP [median score (IQR) 33.8 (15.7–51.7)] (all *p* < 0.001, Table 5).

**Table 5:** Comparison of PREFUL-derived quantitative parameters between the 2 groups.

	All patients (*n* = 77)	CPE group (*n* = 36)	Non-CPE group (*n* = 41)	*P*
Q_N_ (%)	9.3 (6.1–14.2)	6.3 (2.8–9.2)	13.0 (8.8–16.7)	<0.001
QDP (%)	3.5 (2.2–31.7)	33.8 (15.7–51.7)	2.2 (1.4–2.9)	<0.001

Data are provided as medians with 25th and 75th percentiles in parentheses. CPE, Chronic pulmonary embolism; PE, Pulmonary embolism; PREFUL, phase-resolved functional lung; QDP, perfusion defect percentage; Q_N_, normalized perfusion.

## Discussion

In this prospective study, we used PW-PREFUL MRI to detect perfusion defects in patients suspected of having CTEPH. The results were compared with those of V/Q SPECT/CT, revealing substantial agreement between them. Our findings suggested that PW-PREFUL MRI was promising in the clinical evaluation of CPE. Besides, the perfusion parameters derived from PW-PREFUL MRI decreased in patients with CPE.

Early diagnosis is essential in CTEPH, and although lung V/Q scintigraphy is recommended in guidelines, it is not always readily available. CTPA can be used to detect residual emboli; however, sometimes, the imaging characteristics such as eccentric filling defects or the formation of cords or webs in the vessel lumen are subtle. Moreover, whether partial vessel recanalization following thrombosis will result in a perfusion defect remains uncertain. Our study demonstrated that PW-PREFUL MRI exhibited a high performance with a sensitivity of 97%, specificity of 95%, accuracy of 96%, and almost perfect consistency [*κ* = 0.92 (95% CI: 0.83–1.00)] with V/Q SPECT/CT for the diagnosis of CPE at the individual level. Considering that PREFUL MRI is comparable to lung V/Q scintigraphy in detecting pulmonary perfusion defects, it is feasible to provide the perfusion information in the absence of lung V/Q scintigraphy.

Previous studies have reported a substantial agreement (kappa of 0.68) between FD MRI and DCE-MRI in detecting subsegmental and segmental hypoperfusion (30). The diagnostic performance of PW-PREFUL MRI in our study at the segment level was identical to that in previous findings. It suggested that the FD MRI-based approach is quite stable across scanners. Besides, the intraobserver agreement (*κ* = 0.95) and interobserver agreement (*κ* = 0.89) of PW-PREFUL MRI in our study was also almost perfect at the patient level, implying the adequate stability of interpretation.

However, in our study, PREFUL MRI missed a case with CTED, in which SPECT/CT readers classified it as a segmental defect, whereas MRI readers considered it subsegmental. The main cause of 2 false-positive results on PREFUL MRI images was insufficient image quality due to the motion artifacts originating from the heart, resulting in a false perfusion signal. We also observed that the sensitivity for detecting segment perfusion defects was low, which was in accordance with the findings of a recent study (30). This might be attributed to the limited spatial resolution of the PREFUL MRI and increased changes in magnetic susceptibility in the peripheral parts of the lung, which might affect the sensitivity of the method. Nonetheless, our study suggested that the accuracy of PREFUL MRI in detecting segmental defects was higher than in the aforementioned study (30). This might be explained by the less-strict high-pass filtering and additional image-sorting algorithm, which results in more stable image quality, especially for patients with high variability in heart rate. Nevertheless, PREFUL MRI is still limited regarding spatial resolution and might show the artifacts caused by complex cardiac movements nearby, resulting in a lower segmental agreement in the left lung compared with the right lung. These results seem to be in accord with earlier research (31).

In our study, normalized perfusion obtained from PREFUL MRI was reduced in patients with CPE compared with non-CPE patients. Due to the small sample size of patients with CTED, a statistical comparison with CTEPH patients was not possible. According to the results of Pöhler et al. (19), PREFUL MRI may be helpful in the surveillance of therapy effects, especially in the context of CTEPH.

A potential challenge of the routine clinical use of the PREFUL approach was the impact of breathing patterns on imaging. Irregular breathing patterns may widen the spectral lines when applying Fourier analysis, which requires the integration of a larger frequency range, leading to more noise being added to the calculated images (14). Hence, irregular breathing can lead to a low signal-to-noise ratio and may affect the quality of ventilation- and perfusion-weighted maps. Moreover, irregular breathing may affect the variability of ventilation measurement (15, 32). In order to deal with nonstationary signals, different methods developed to achieve a more robust post-processing algorithm such as wavelet analysis, adapted filter design, and an optimized registration to account for large deformation steps could be beneficial (33).

Also, other challenges for the application of the PREFUL approach are the variability of quantified perfusion, which is affected by various technical and physiological conditions, such as high signal variability in the completely blood-filled voxels, gadolinium contrast administration, field inhomogeneities, physiological perfusion variability, inaccuracy of the estimated receive coil sensitivities or the direction of blood flow toward the imaging plane (17, 34, 35). In our study, signal variability inside blood-filled ROI may be potentially corrected by the automated PREFUL postprocessing algorithm (17).

More and more 3 T scanners are being applied in clinical practice, and it is important to select an appropriate sequence for the PREFUL method. Many studies have shown that an optimized spoiled gradient echo (SPGR) sequence is feasible at 1.5 T or 3 T, while a bSSFP sequence is limited at 3 T compared to 1.5 T (20, 35, 36). Because SPGR is less prone to susceptibility artifacts and uses small flip angles, it might be advantageous for PREFUL at 3 T. In addition, field strength can also affect the PREFUL-derived perfusion parameters. Glandorf et al. revealed a significant 27% decrease in quantified perfusion at 3 T due to an increase in the T1 relaxation time of blood in the lungs on higher field strengths, and a significant 38% decrease in QDP at 3 T, which may be related to field inhomogeneities (35). Given that the technical conditions and physiological factors lead to limited comparability of PREFUL quantitative parameters, more fully quantified biomarkers need to be developed in further studies to achieve optimal comparability.

This study had certain limitations. First, the sample size was limited to 77 patients including 31 with CTEPH and 5 with CTED. These data indicated that our study was a monocentric study in a PH referral center, and the positive rate of CTEPH was relatively high. Thus, the positive predictive value might only be effective in populations with a high likelihood of having CTEPH. Considering its high sensitivity and specificity, MRI would be expected to perform well in patients with persistent or new-onset dyspnea after acute PE having risk factors for CTEPH. Second, we were unable to perform the right cardiac catheterization in all patients. This was because a few patients could not undergo further right heart catheterization (RHC) when V/Q scanning was negative, leading to a gold standard bias. However, V/Q SPECT is the most recommended screening protocol in routine clinical practice and may not be problematic. Third, the ventilation imaging in PREFUL MRI was not included for analysis in this study. Although ventilation imaging is necessary for the diagnosis of PE, it has been replaced by the UTE protocol because the latter can evaluate lung parenchyma like CT (37). Previous literature has demonstrated either substantial or almost perfect agreements between the two methods (38), rendering the single MRI examination as comprehensive as a V/Q SPECT/CT. Fourth, although the CT examination in SPECT and the UTE sequence of PREFUL MRI can be used for lung segment localization, resolution-limited fine and accurate segmentation of lung segments might be a problem of this study. In addition, the standardized protocols for acquiring consistent and reproducible perfusion images are lacking. Further studies are required to overcome the aforementioned limitations and validate the role of PREFUL in CPE.

Despite the mentioned limitations, PREFUL MRI provides a valuable and attractive alternative imaging method, without the need for additional expensive equipment and hard-to-get gaseous tracers. It is particularly suitable for patients who cannot hold their breath and those who cannot accept contrast agents, making it helpful for pregnant women, children, and patients with kidney failure. In addition, more robust biomarkers were obtained by applying automated PREFUL MRI postprocessing methods into the clinical workflow, which can monitor different lung diseases without the risk of radiation. Furthermore, functional PREFUL MRI in combination with morphological sequences may be a great advantage over other techniques.

## Conclusion

PREFUL MRI is a promising tool to assess CPE/CTEPH during free breathing without the use of ionizing radiation or contrast agents, providing a second-line alternative to SPECT/CT.

## Data availability statement

The raw data supporting the conclusions of this article will be made available by the authors, without undue reservation.

## Ethics statement

This study received approval from the China-Japan Friendship Hospital Ethical Review Board (Medical Ethics No. 2017-24). The studies were conducted in accordance with the local legislation and institutional requirements. The participants provided their written informed consent to participate in this study. Written informed consent was obtained from the individual(s) for the publication of any potentially identifiable images or data included in this article.

## Author contributions

JD: Conceptualization, Data curation, Formal analysis, Investigation, Project administration, Validation, Visualization, Writing – original draft. SX: Conceptualization, Supervision, Writing – review & editing. HS: Formal analysis, Writing – review & editing. JA: Writing – review & editing, Software. HL: Writing – review & editing, Formal analysis. LL: Formal analysis, Writing – review & editing. RG: Writing – review & editing, Software. AV: Software, Writing – review & editing. JV-C: Software, Writing – review & editing.

## Funding

The authors declare that no financial support was received for the research, authorship, and/or publication of this article.

## Conflict of interest

JA and RG are employees of Siemens Healthineers. AV is an employee of BioVisioneers GmbH, who holds a patent for the Method of quantitative magnetic resonance lung imaging (Patent number: 10010293).

The remaining authors declare that the research was conducted in the absence of any commercial or financial relationships that could be construed as a potential conflict of interest.

## Publisher’s note

All claims expressed in this article are solely those of the authors and do not necessarily represent those of their affiliated organizations, or those of the publisher, the editors and the reviewers. Any product that may be evaluated in this article, or claim that may be made by its manufacturer, is not guaranteed or endorsed by the publisher.
